# Sarcopenia’s Prognostic Impact on Patients Treated with Immune Checkpoint Inhibitors: A Systematic Review and Meta-Analysis

**DOI:** 10.3390/jcm10225329

**Published:** 2021-11-16

**Authors:** Donggun Lee, Na Won Kim, Jong Yeob Kim, Joo Hyung Lee, Ji Hyun Noh, Haejun Lee, Jin Woon Jeong, Seungeun Lee, Jeonghyun Kang

**Affiliations:** 1 Department of Surgery, Gangnam Severance Hospital, Yonsei University College of Medicine, Seoul 06273, Korea; bnbn1245@gmail.com (D.L.); ristar1984@yuhs.ac (J.H.L.); NJH119@yuhs.ac (J.H.N.); YADUN87@yuhs.ac (H.L.); JJW115@yuhs.ac (J.W.J.); LSE9412@yuhs.ac (S.L.); 2 Medical Library, Yonsei University College of Medicine, Seoul 03722, Korea; nwkim@yuhs.ac; 3 Yonsei University College of Medicine, Seoul 03722, Korea; crossing96@yonsei.ac.kr

**Keywords:** sarcopenia, immune checkpoint inhibitors, hazard ratio, overall survival, progression-free survival

## Abstract

Background: Although sarcopenia has been reported to predict survival in cancer patients, its impact on patients who received immune checkpoint inhibitors (ICIs) has not been thoroughly investigated. This systematic review aimed to assess the long-term oncologic impact of sarcopenia on patients who received ICIs. Methods: A systematic review of studies indexed in the PubMed, Embase, and Cochrane databases, up to April 1, 2021, was conducted. Studies that reported hazard ratios (HRs) for overall survival (OS) and progression-free survival (PFS) based on sarcopenia in patients treated with ICIs were included. The inverse variance method was used with a random-effects model for data analysis. Results: A total of 1284 patients from 14 studies were included. Among the patients who received ICIs, patients with sarcopenia had a significant increase in overall mortality compared to patients without sarcopenia in univariate analyses (HR = 1.66, 95% CI = 1.20–2.29, *p* = 0.002) and in adjusted HRs (HR = 1.55, 95% CI = 1.15–2.10, *p* = 0.004). The same results were obtained for PFS by both univariate analysis (HR = 1.75, 95% CI = 1.37–2.23, *p* < 0.001) and adjusted HRs (HR = 1.63, 95% CI 1.28–2.09, *p* < 0.001). Conclusions: Sarcopenia appears to be an effective biomarker for predicting long-term oncologic outcomes in patients receiving ICI therapy and hence plays an important role when making treatment decisions. However, the fundamental role of this association with survival should be further investigated in large cohorts and clinical trials.

## 1. Introduction

Sarcopenia and changes in muscle mass during a certain treatment period have been evaluated as important prognosticators in cancer patients [1,2,3,4,5]. The clinical importance of myosteatosis, which indicates the mean Hounsfield Unit of skeletal muscle measured using computed tomography (CT), has also been studied, and its association with oncologic outcomes has also been determined in patients with various types of cancers [6,7].

Immunotherapy with immune checkpoint inhibitors (ICIs) has become one of the major breakthroughs in advanced cancers [8]. The clinical benefits and side effects of ICIs vary, and they include hyperprogression among patients with cancer [9,10,11]. Therefore, investigating the biomarkers relevant to ICI response is important for predicting patients’ clinical outcomes. Recently, the clinical significance of sarcopenia in patients who have undergone ICI therapy has been reported. Cortellini et al. reported that a low skeletal muscle index (SMI) was associated with poor oncologic outcomes in advanced cancer patients treated with ICIs [12]. Shimizu et al. reported that the psoas muscle index (PMI) might be a significant prognostic factor for progression-free survival (PFS) and overall survival (OS) following ICI therapy for metastatic urothelial carcinoma [13]. In contrast, Minami et al. observed no significant correlation between sarcopenia and clinical outcomes in patients treated with ICIs [14]. To incorporate these findings and investigate the role of sarcopenia as a possible prognostic factor in patients who underwent ICIs, a review of the existing evidence seemed appropriate. Although a recent meta-analysis reported that sarcopenia could be used as a viable option for predicting prognosis in non-small cell lung cancer (NSCLC) patients who received ICIs [15], the impact of sarcopenia has not been thoroughly investigated in patients with other types of cancer. 

Therefore, we performed a systematic review and meta-analysis to evaluate the long-term effects of sarcopenia on survival in cancer patients treated with ICIs.

## 2. Materials and Methods

### 2.1. Search Strategy

This systematic review was performed according to the Preferred Reporting Items for Systematic Reviews and Meta-Analyses (PRISMA) guidelines [16]. We performed computerized bibliographic searches from inception to April 2021. The detailed search strategies and associated lists following PubMed/Medline, Embase, and Cochrane library databases were extracted by one of our authors (N.W.K.) and are included in the Supplementary Tables S1 and S2. Manual searching of reference lists from the included articles was also performed to identify additional potential results.

### 2.2. Study Selection and Data Extraction

The search identified 1706 articles. Two authors (D.L. and J.K.) screened all the titles and abstracts of the identified articles. After screening, full-text reviews were independently performed by the authors to determine the eligibility of the studies. Discrepancies were resolved through discussion. Studies were selected based on several inclusion criteria. We defined the study population as various types of cancer patients who underwent ICI therapy. The primary outcomes were measured as hazard ratios (HRs) of OS and/or PFS with sarcopenia as one of the variables. In general, the previously established definition of sarcopenia is what was most commonly used; however, some studies employed newly defined criteria for sarcopenia, which were also included in our study. Conference abstracts, editorials, comments, and publications published in languages other than English were not eligible for inclusion.

Two authors (D.L. and J.K.) extracted data details on authors, year, publication country, cancer type, stage, time point of CT examination, ICI type, measurement of sarcopenia, cut-off value of sarcopenia, number of included patients, and number and proportion of sarcopenia patients. Any discrepancies in study selection and data extraction between the two authors were resolved by discussion.

### 2.3. Study Quality Assessments

The risk of bias and quality of individual studies were assessed using the Newcastle-Ottawa Scale (NOS) [17]. The NOS uses three categories with each item: (1) selection (representativeness of the exposed cohort, selection of the non-exposed cohort, ascertainment of exposure, and demonstration that the outcome of interest was not present at the start of the study); (2) comparability (comparability of cohorts based on the design or analysis); and (3) outcome (assessment of outcome, follow-up long enough for outcomes to occur, and adequacy of follow-up of cohorts). A study could be awarded a maximum of one point for each numbered item within the selection and outcome categories. A maximum of two points could be given for comparability. The NOS scores were up to nine points. A study that scored 7 or higher points was considered to be of high quality, 5–6 points moderate quality, and below 4 points low quality. 

In addition, we used the Grading of Recommendations, Assessment, Development and Evaluation (GRADE) system to assess the certainty of the evidence for each outcome of our meta-analysis [18]. The GRADE system consists of study limitations (risk of bias), inconsistency, indirectness, imprecision, and publication. For our observational study, the GRADE system also considers a large effect, plausible confounding, and the dose-response gradient. Based on these categories, two authors classified the certainty of the results as high, moderate, low, or very low.

### 2.4. Statistical Analysis

The effects of sarcopenia on outcomes were measured using the HRs and CIs of OS and PFS. Meta-analysis was performed using the two different outcomes, such as HRs in univariate analysis and adjusted HRs, of each individual studies. Pooled HRs with 95% CIs indicated the effects of sarcopenia on outcomes. A Cochran Q statistic *p*-value < 0.05 or an I^2^ statistic > 50% were evaluated to represent statistically significant heterogeneity between the studies. A random-effects model was used to pool the HRs. Inverse-variance weighting was used to pool estimates from the included studies. While measuring the heterogeneity between the included studies, we excluded one study because it used the HR of sarcopenia as a continuous variable [19]. Subgroup analyses were conducted according to the type of cancer, receipt of different types of ICIs, or different definitions of sarcopenia. Funnel plots and Egger linear regression analysis were not possible because fewer than ten studies were included.

The meta-analysis was performed using Review Manager software (RevMan, Version 5.41, for Windows, Oxford, UK; the Cochrane Collaboration, 2014). All *p*-values were two-sided, and except for the test of discrepancy, the *p*-value < 0.05 was considered statistically significant.

## 3. Results

### 3.1. Search Results and Study Population Characteristics

**Figure 1:**
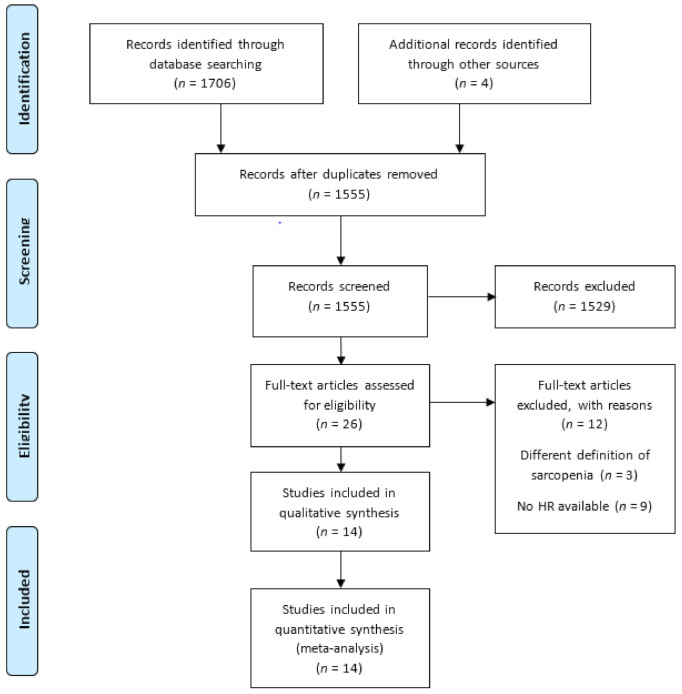
Flow diagram of the study selection process. Abbreviation: HR, hazard ratios.

The results of the PRISMA flow diagram are shown in Figure 1. Our initial database searches and manual searches identified 1710 articles. After the duplicates were removed, 1555 articles remained. We then examined the titles and abstracts, and 1529 articles were excluded. We then performed full-text reviews based on the inclusion criteria. We excluded articles that defined sarcopenia as differences in SMI between pre-treatment and post-treatment [20,21,22] or those that did not show HRs of OS and/or PFS [23,24,25,26,27,28,29,30,31]. Finally, 14 studies with a total of 1284 patients were included in this study [12,13,14,19,32,33,34,35,36,37,38,39,40,41].

The details of the included studies are presented in Table 1. All the included studies had a retrospective design. No prospective studies were available. All the included studies were published after 2019. The majority of the cancer types in the included studies were NSCLC (7), followed by hepatocellular carcinoma (2), melanoma (2), urothelial carcinoma (2), renal cell carcinoma, and gastric cancer. In terms of the types of ICIs, 13 studies used anti-PD-1/PD-L1 ICIs, and only one study used anti-CTLA-4. The cut-off values and sarcopenia reference varied across studies. SMI was used to define sarcopenia in 10 studies, whereas PMI was used to measure sarcopenia in 4 studies. Result types of the studies included in the meta-analysis are shown in Supplementary Table S3.

### 3.2. Primary Outcome

**Figure 2:**
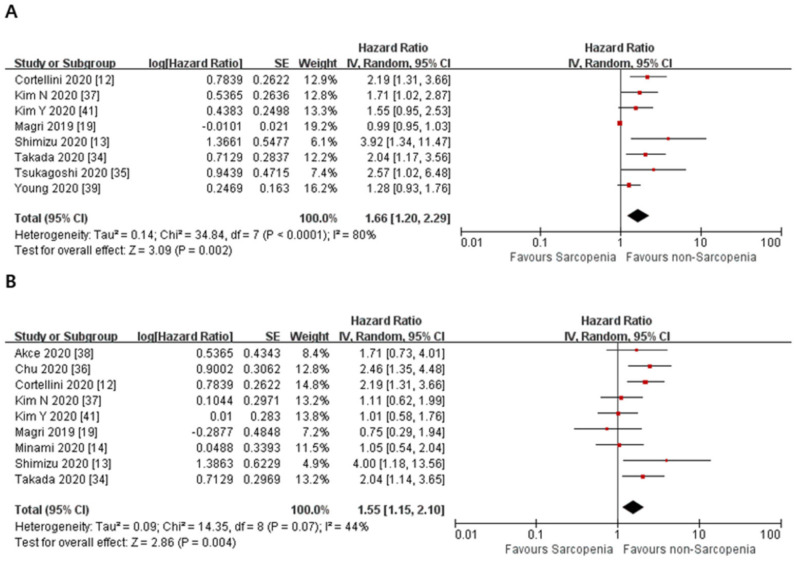
Forest plots of hazard ratios of sarcopenia on overall survival in cancer patients treated with immune checkpoint inhibitors. (**A**) Overall survival in the univariate analysis. (**B**) Overall survival with adjusted HRs.

**Figure 3:**
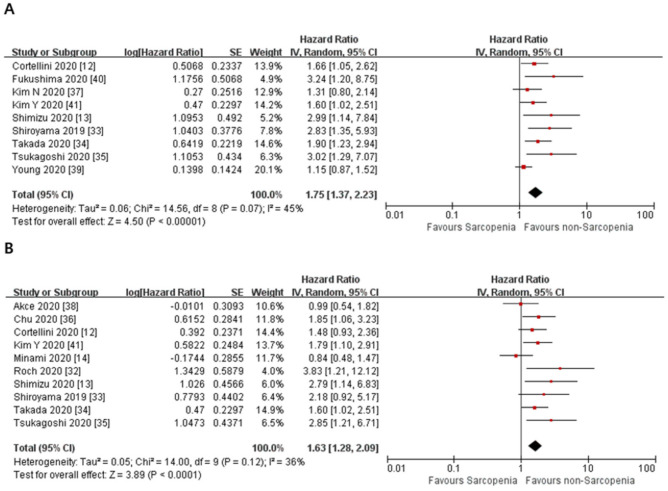
Forest plots of hazard ratios of sarcopenia on progression-free survival in cancer patients treated with immune checkpoint inhibitors. (**A**) Progression-free survival in the univariate analysis. (**B**) Progression-free survival with adjusted HRs.

The meta-analysis of the included studies showed the pooled HR of 1.66 for OS in the studies that used univariate analysis (95% CI = 1.20–2.29, *p* = 0.002). The heterogeneity among these studies was significant (Cochran’s Q statistic, *p* < 0.0001, I^2^ = 80%). For the studies that used adjusted HRs, the meta-analysis of the included studies showed the pooled HR of 1.55 for OS (95% CI = 1.15–2.10, *p* = 0.004). The heterogeneity among these studies was not significant (Cochran’s Q statistic, *p* = 0.07, I^2^ = 44%) (Figure 2). In addition, we performed a meta-analysis of PFS. For the studies that used univariate analysis, the pooled HR was 1.75 for PFS (95% CI = 1.37–2.23, *p* < 0.00001). The heterogeneity among these studies was not significant (Cochran’s Q statistic, *p* = 0.07, I^2^ = 45%). For the studies that used adjusted HRs, the pooled HR was 1.63 for PFS (95% CI = 1.28–2.09, *p* < 0.0001). The heterogeneity among these studies was also not significant (Cochran’s Q statistic, *p* = 0.12, I^2^ = 36%) (Figure 3).

### 3.3. Subgroup Analyses

To investigate the cause of the heterogeneity in the OS in the studies using HRs extracted from univariate analysis, we excluded the study by Magri et al., which expressed the HR of sarcopenia uniquely as a continuous variable. After excluding that study, the heterogeneity decreased in the studies using HRs extracted from the univariate analysis of OS (HR = 1.74, 95% CI = 1.39–2.19, *p* < 0.00001, Cochran Q statistic, *p* = 0.27, I^2^ = 21%) and the studies using adjusted HRs of OS (HR = 1.64, 95% CI = 1.21–2.21, *p* = 0.001, Cochran Q statistic, *p* = 0.10, I^2^ = 41%) (Supplementary Figure S1). 

We performed subgroup analysis according to cancer type. Because of the study number, only OS of the studies with adjusted HRs were eligible (Supplementary Figure S2). The test for subgroup differences suggested that there was no statistically significant subgroup effect (*p* = 0.42, I^2^ = 0%), implying that cancer type does not modify the effect of ICIs on OS in different cancer types. However, this analysis may not be able to detect subgroup differences due to the discrepancies in the study numbers and participant numbers. In the case of the studies derived from univariate analysis of PFS, the test for subgroup differences suggested that there was a statistically significant subgroup effect (*p* = 0.05, I^2^ = 81.0%) (Supplementary Figure S3). 

We performed subgroup analysis by type of ICI using the HRs of PFS. The test for sub-group differences suggested that there was no statistically significant subgroup effect (*p* = 0.22, I^2^ = 34.4%) (Supplementary Figure S4).

In this meta-analysis, either SMI or PMI was used to define sarcopenia. Comparing SMI and PMI, a significant subgroup effect was observed in the studies using univariate analysis of OS (*p* = 0.06, I^2^ = 71.0%); however, there was no statistically significant subgroup effect (*p* = 0.80, I^2^ = 0%) OS of the studies using adjusted HRs (*p* = 0.80, I^2^ = 0%) (Supplementary Figure S5). With respect to PFS, there was a statistically significant subgroup effect (*p* = 0.01, I^2^ = 83.2%) in the studies using univariate analysis of PFS, whereas no statistically significant subgroup effect was observed in the PFS of the studies using adjusted HRs (*p* = 0.69, I^2^ = 0%) (Supplementary Figure S6). 

### 3.4. Quality Assessment

We used the NOS to assess the risk of bias and the quality of the individual studies. Of the 14 studies, five were assessed as high quality and nine as moderate quality. Detailed assessments are presented in Table 2. We then used the GRADE system to assess the certainty of the evidence in our meta-analysis. The results of the GRADE certainty assessment for the meta-analysis of each outcome are presented in Table 3. We are very uncertain about the prognostic effect of sarcopenia in cancer patients treated with ICIs.

## 4. Discussion

This systematic review and meta-analysis evaluated the prognostic effects of sarcopenia in patients with ICIs. The meta-analysis of the included studies showed poor prognosis of patients with sarcopenia with respect to OS (HR, 1.55; 95% CI = 1.15–2.10, *p* = 0.004 for OS using studies with adjusted HRs) and PFS (HR, 1.75; 95% CI = 1.37–2.23, *p* < 0.001 for PFS using studies with adjusted HRs). 

Since ICIs were introduced as an alternative treatment option in various advanced and refractory cancer patients [42], identifying the biomarkers relevant to ICI outcomes has been actively investigated. Higher tumor mutational burden, microsatellite instability, and PD-L1 immunohistochemical staining have been proven to be strong predictive markers for better responses [43]. However, these biomarkers are difficult to use because additional laborious work or obtaining adequate tissue is required. Recently, a growing body of evidence has reported that patient host factors, such as body composition, are associated with the clinical efficacy of ICIs. Sarcopenia, which was initially defined as age-associated loss of muscle mass in elderly persons [44], has been incorporated into the oncology field, and the prognostic impact of sarcopenia or myosteatosis in cancer patients treated with surgery and/or palliative or adjuvant chemotherapy has been well studied [1,6,7]. Several retrospective studies have explored the impact of sarcopenia on ICIs, and most of them were included in this meta-analysis. Most patients diagnosed with a certain type of cancer underwent abdominopelvic CT to determine the extent of the disease at the initial stage. The lack of additional demand to assess sarcopenia is a very important advantage in terms of clinical use.

The impact of sarcopenia on ICIs can be explained in several ways. Chronic inflammation in cancer, a major contributor to the sarcopenia cascade [45], causes immune dysfunction, such as T cell exhaustion, which is characterized by a loss of effector function, prolonged and high expression of multiple inhibitory receptors, and specific transcriptional pathways [46]. It is mediated by changes in the functions of cytokines and results in a reduced response of ICIs. In addition, skeletal muscle tissue synthesizes cytokines and other proteins. They are collectively called myokines. Myokines, such as IL-6, IL-15, TNF-α, and TGF-β, exert autocrine, endocrine, and paracrine effects on many tissues. With altered activities of these myokines in the setting of sarcopenia, the immune system leans towards exhibiting pro-inflammatory effects and muscle catabolism, as well as inducing immune senescence [47,48]. Additionally, the role of gut microbiome in developing sarcopenia and modulating ICIs’ responses was recently introduced. The gut microbiome is extensively involved in the immune system by anatomic features and the need to modulate the numerous variant species in the gastrointestinal tract. In patients with altered gut microbiome, the gut dysbiosis may result in promoting a pro-inflammatory pathway related to sarcopenia. However, the pathophysiology of the role of gut microbiome to regulate the response to ICIs was not fully investigated yet [49,50].

Whether sarcopenia could be used as a predictive marker for immune-related adverse events (irAEs) remains unclear. A recent meta-analysis by Wang et al. reported that sarcopenia was not associated with an increased rate of irAEs (relative risk = 0.99, 95% CI = 0.21–4.67) in patients with NSCLC [15]. Another systematic review reported that sarcopenia was correlated with adverse events; however, no association with increased irAEs was noted [51]. Therefore, it is unclear whether poor oncologic outcomes for sarcopenic patients are directly derived from ICI-induced toxicities or reduced adherence to ICI treatments. Further research needs to be done to reveal the fundamental mechanism of this correlation.

Our study had some limitations. The included studies were mainly retrospective in design, with a relatively small number of included patients. The inevitable selection bias of the original studies might be one of the reasons for the heterogeneity. Additionally, we could not adjust covariates equally due to the different variables used in each included study, which are shown in Supplementary Table S4. This situation caused heterogeneity, which resulted in increased values of Q-statistic’s *p*-value and I-square. Thus, we estimated a pooled HR considering these variabilities via the random effect model. We excluded several studies that did not provide adequate survival outcomes, such as HRs and 95% CI. Notably, there were severe methodological variations in defining sarcopenia and its cut-off values across the studies in this meta-analysis. Most of the included studies used the SMI cut-off value suggested by Martin et al. However, PMI was alternatively used because of its potential ease of measurement. Studies comparing the skeletal muscle area (SMA) and psoas muscle area (PMA) showed discordant results. Rutten et al. reported a weak correlation (Pearson correlation of 0.52) between the SMA and PMA in ovarian cancer patients [52], whereas Jones et al. reported a better correlation (Spearman correlation of 0.8) in patients with colorectal cancer [53]. The reason for this discrepancy might be explained by different statistical methods, sex differences, and the relatively small number of patients. When we tried to measure the effect size, these different definitions caused heterogeneity and other unexpected biases. Based on the subgroup analysis according to the different methods used to define sarcopenia, some subgroup differences were observed between SMI and PMI with respect to the studies from the univariate analysis of OS and PFS. Nevertheless, there was no difference in the overall trends favoring a better prognosis in patients without sarcopenia. Although the lack of a consensus on the definition of sarcopenia may hamper the wide application of our observations in clinical practice, our results show the possibility that PMI can be used to predict sarcopenia in patients receiving ICIs. Additionally, we could not exclude publication bias where studies of opposite results were not published. We could not use a funnel plot to distinguish real asymmetry due to the small sample sizes. Finally, the measured time gap between CT examination and treatment initiation showed a huge diversity, ranging from 1 month to 6 months across the included studies. Considering malnourishment and advanced tumor stage, the time gap might be one of the critical determinants of sarcopenia. The different time gaps between studies may hinder the standardization of this parameter. For future studies dealing with this issue, these limitations should be considered.

## 5. Conclusions

Our study investigated the effect of sarcopenia on clinical outcomes across all types of cancer in patients treated with ICIs. The results suggested that sarcopenia could negatively affect OS and PFS in patients with cancer who were treated with ICIs in both univariate and with adjusted HRs. Sarcopenia could be used as a marker for predicting ICI clinical outcomes and needs to be assessed before treatment with ICIs.

## Figures and Tables

**Table 1:** Characteristics of the studies included in the meta-analysis.

Author, Year	Country	Cancer	Stage	Time Point of CT Exam ^a^	ICI Type	Measurement of Sarcopenia	Cut-Off Value of Sarcopenia ^b^	No. of Patients	Median Age of Patients	No. of Sarcopenia(%)
Minami 2020 [14]	Japan	NSCLC	Advanced	90 days	Nivolumab, Pembrolizumab, Atezolizumab	PMI	Male:6.36, Female:3.92	74	70	53(71)
Magri 2019 [19]	Italy	NSCLC	Stage IV	10 weeks	Nivolumab	SMI	NA	46	65	NA
Roch 2020 [32]	France	NSCLC	Metastatic	NA	Nivolumab, Pembrolizumab	SMI	Male: 52.4, Female: 38.5	142	64	92(66)
Shiroyama 2019 [33]	Japan	NSCLC	Advanced	90 days	Nivolumab, Pembrolizumab	PMI	Male:6.36, Female:3.92	42	71	22(52)
Takada 2020 [34]	Japan	NSCLC	Stage III, IV/Recurred	NA	Nivolumab, Pembrolizumab	SMI	Male: 25.63, Female: 21.73	103	67	51(49)
Tsukagoshi 2020 [35]	Japan	NSCLC	Stage III, IV	30 days	Nivolumab	PMI	Male:6.36, Female:3.92	30	67	13(43)
Akce 2020 [36]	USA	HCC	Advanced	2 months	Anti PD-1, Anti-PD-1 + others(not specified)	SMI	Male: 43, Female: 39	57	66	28(49)
Kim N 2020 [37]	Korea	HCC	Advanced	NA	Nivolumab	SMI	Male: 42, Female: 38	102	61	23(23)
Chu 2020 [38]	Canada	Melanoma	Metastatic/ advanced	30 days	Ipilimumab	SMI	Male: 43(52 ^c^), Female: 41	97	56	NA
Young 2020 [39]	USA	Melanoma	Metastatic/ advanced	6 months	Nivolumab, Pembrolizumab, Atezolizumab, Ipilimunab + nivolumab	SMI	Male: 43(52 ^c^), Female: 41	287	63	154(54)
Shimizu 2020 [13]	Japan	Urothelial carcinoma	Metastatic/ advanced	NA	Pembrolizumab	PMI	Male:6.36, Female:3.92	27	73	15(56)
Fukushima 2020 [40]	Japan	Urothelial carcinoma	Advanced	30 days	Pembrolizumab	SMI	Male: 43(52 ^c^), Female: 41	28	74	19(68)
Kim Y 2020 [41]	Korea	Gastric cancer	Metastatic	3 months	Nivolumab, Pembrolizumab	SMI	Male: 49, Female: 31	149	57	79(53)
Cortellini 2020 [12]	Italy	NSCLC, Melanoma, RCC, others	Advanced	90 days	Pembrolizumab, Nivolumab, Atezolizumab, and others	SMI	Male: 48.4(50.2 ^c^), Female: 36.9(59.6 ^c^)	100	66	50(50)

Abbreviations: ICI, immune-checkpoint inhibitor; NSCLC, non-small cell lung cancer; HCC, hepatocellular carcinoma; NA, not available; SMI, skeletal muscle index; PMI, psoas muscle index; ^a^: time within initiation of ICI therapy; ^b^: cm^2^ /m^2^; ^c^: patient with body mass index >25kg/m^2.^

**Table 2:** The Newcastle-Ottawa Scale (NOS) of the studies included in the meta-analysis.

	Selection				Comparability	Outcome			Total
Author, Year	Representativeness of the Exposed Cohort	Selection of the Non-Exposed Cohort	Ascertainment of Exposure	Demonstration That Outcome of Interest Was Not Present at Start of Study	Comparability of Cohorts on the Basis of the Design or Analysis ^a^	Assessment of Outcome	Was Follow-Up Long Enough for Outcomes to Occur ^b^	Adequacy of Follow Up of Cohorts ^c^	
Young 2020 [39]	1	1	1	1	0	1	1	1	7
Minami 2020 [14]	1	1	1	1	0	1	1	1	7
Shimizu 2020 [13]	1	1	1	1	0	1	1	1	7
Shiroyama 2019 [33]	1	1	1	1	0	1	1	1	7
Kim Y 2020 [41]	1	1	1	1	0	1	1	1	7
Cortellini 2020 [12]	1	1	1	1	0	1	1	0	6
Roch 2020 [32]	1	1	1	1	0	1	1	0	6
Fukushima 2020 [40]	1	1	1	1	0	1	1	0	6
Takada 2020 [34]	1	1	1	1	0	1	1	0	6
Akce 2020 [36]	1	1	1	1	0	1	1	0	6
Chu 2020 [38]	1	1	1	1	0	1	0	1	6
Kim N 2020 [37]	1	1	1	1	0	1	1	0	6
Magri 2019 [19]	1	1	1	1	0	1	1	0	6
Tsukagoshi 2020 [35]	1	1	1	1	0	1	0	0	5

^a^: None of the included studies adequately controlled confounders. ^b^: If individual studies specified follow-up duration, we assessed long enough for outcomes to occur. ^c^: If individual studies lost more than 30% of the included patients or did not specify those lost, we assessed as inadequate.

**Table 3:** Results of GRADE certainty assessment for evidence of impact of sarcopenia in patients treated with immune checkpoint inhibitors.

Certainty Assessment							Effect	Certainty	
No of Studies	Study Design	Risk of Bias	Inconsistency	Indirectness	Imprecision	Other Considerations	Relative (95% CI)		
Overall survival in univariate analysis									
8	observational studies	serious ^a^	not serious ^b^	not serious	not serious	none	HR 1.66 (1.20 to 2.29)	⨁◯◯◯ VERY LOW	
Overall survival with adjusted HRs									
9	observational studies	serious ^a^	not serious	serious ^c^	not serious	none	HR 1.55 (1.15 to 2.10)	⨁◯◯◯ VERY LOW	
Progression free survival in univariate analysis									
9	observational studies	serious ^a^	not serious	not serious	not serious	none	HR 1.75 (1.37 to 2.23)	⨁◯◯◯ VERY LOW	
Progression free survival with adjusted HRs									
10	observational studies	serious ^a^	not serious	serious ^c^	not serious	none	HR 1.63 (1.28 to 2.09)	⨁◯◯◯ VERY LOW	

Explanations: CI: confidence interval; HR: hazard ratio; ^a^. Failure to adequately control confounding; ^b^. Not downgraded for inconsistency despite substantial heterogeneity given its likely not clinical meaningful; ^c^. Indirectness on the outcome in concept of adjusted HRs.

## Data Availability

Not applicable.

## Supplementary Materials

The following are available online at https://www.mdpi.com/article/10.3390/jcm10225329/s1. Supplementary Figure S1. Forest plots of hazard ratios of overall survival according to the sarcopenia, excluding Magri et al.’s study. Supplementary Figure S2. Forest plots of hazard ratios for overall survival with adjusted HRs by cancer type. Supplementary Figure S3. Forest plots of hazard ratios for progression-free survival in the univariate analysis by cancer type. Supplementary Figure S4. Forest plots of hazard ratios for progression-free survival in the univariate analysis according to the type of immune checkpoint inhibitors. Supplementary Figure S5. Forest plots of hazard ratios for overall survival by skeletal muscle index and psoas muscle index. Supplementary Figure S6. Forest plots of hazard ratios for progression-free survival by skeletal muscle index and psoas muscle index. Supplementary Table S1. Search strategy. Supplementary Table S2. Search query and number of items found. Supplementary Table S3. Result types of the studies included in the meta-analysis. Supplementary Table S4. Covariates of the studies using adjusted HRs.
