# Supplementary material for: Sarcopenia’s Prognostic Impact on Patients Treated with Immune Checkpoint Inhibitors: A Systematic Review and Meta-Analysis
Source: J Clin Med. 2021 Nov 16;10(22):5329. doi: 10.3390/jcm10225329 (PMC8622936; doi:10.3390/jcm10225329)
Supplement: Supplementary file 1 [file jcm-10-05329-s001.zip › jcm-1410596-supplementary.pdf]

Journal of
Clinical Medicine

MDPI

Supplementary File

Sarcopenia's prognostic impact on patients treated with immune checkpoint inhibitors: a systematic review and meta-analysis

A

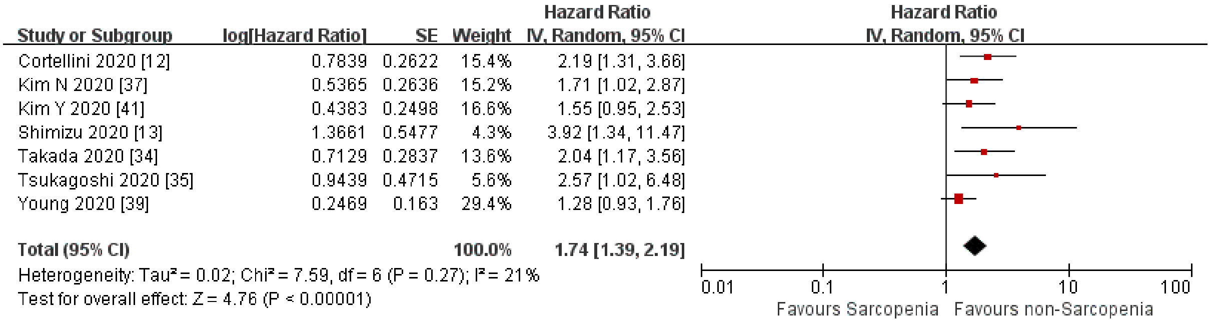

B

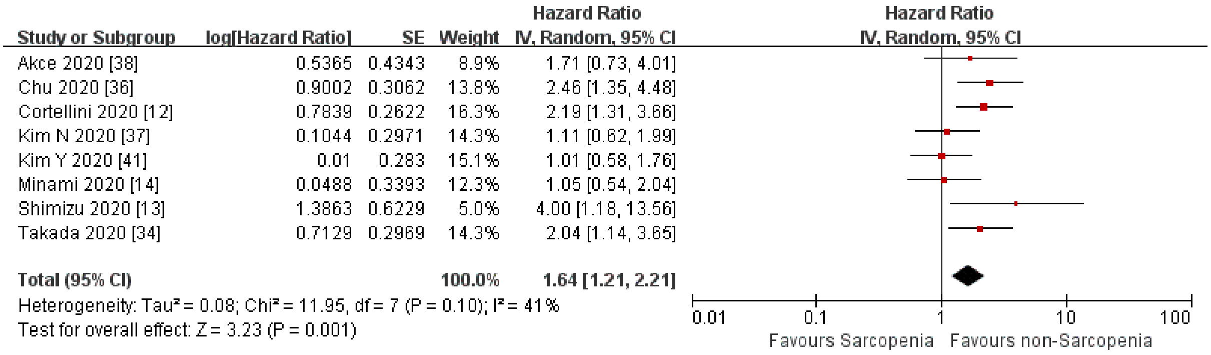

Supplementary Figure S1. Forest plots of hazard ratios of overall survival according to the sarcopenia excluding Magri et al. study—(**A**) overall survival in the univariate analysis, (**B**) overall survival with adjusted HRs.

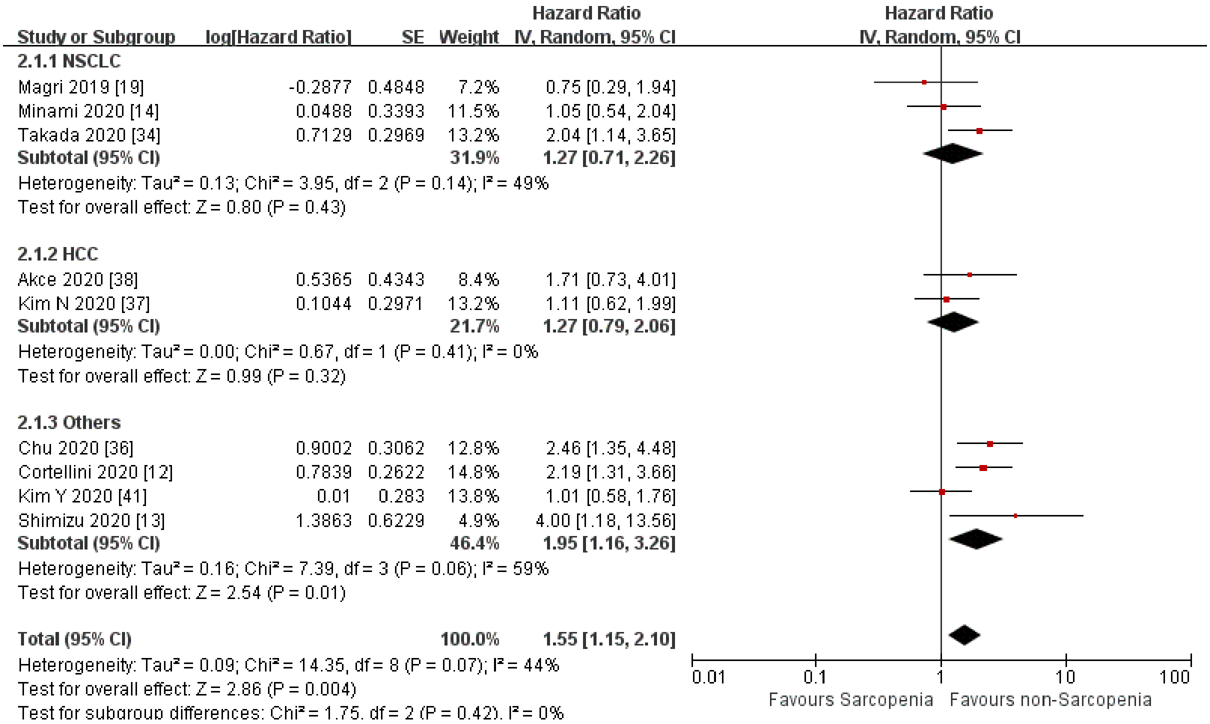

Supplementary Figure S2. Forest plots of hazard ratios for overall survival with adjusted HRs by cancer type.

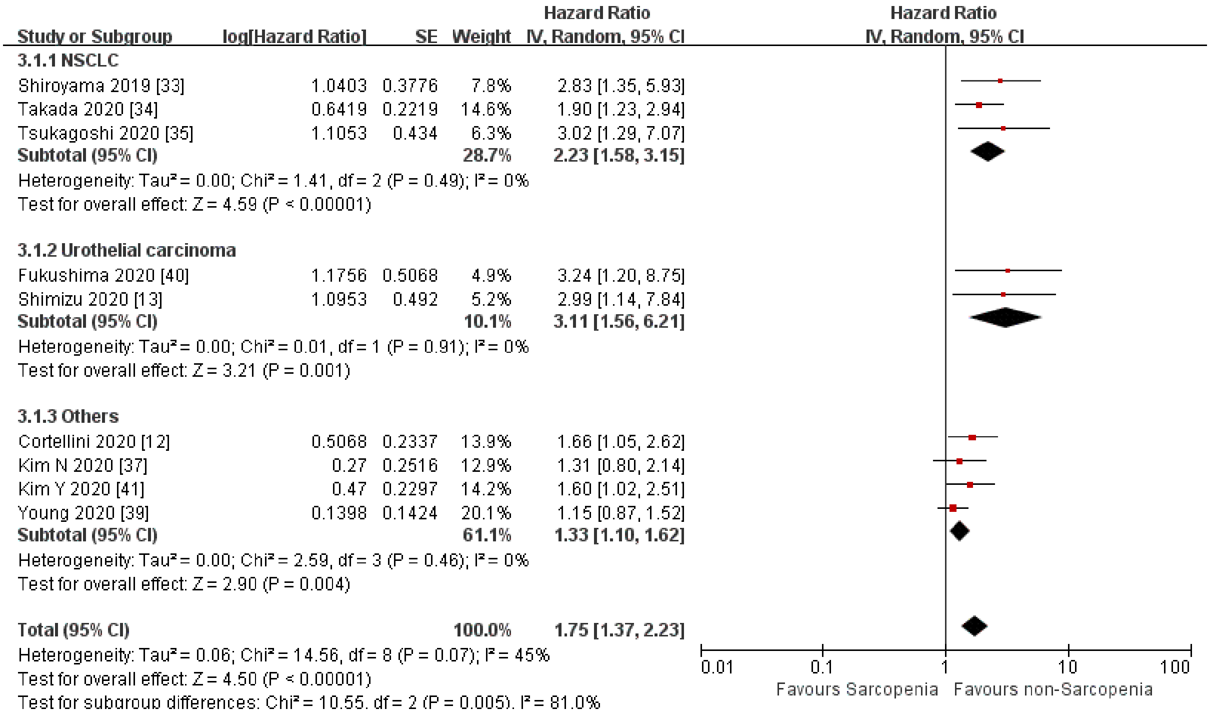

Supplementary Figure S3. Forest plots of hazard ratios for progression-free survival in the univariate analysis by cancer type.

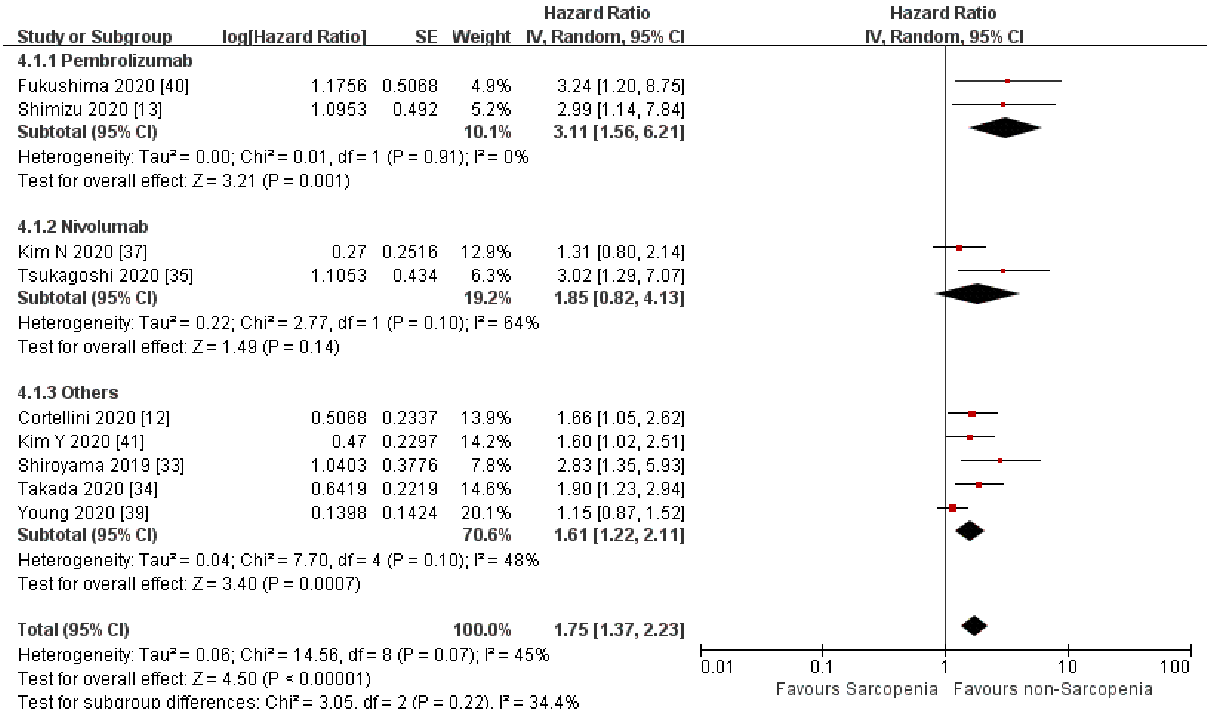

Supplementary Figure S4. Forest plots of hazard ratios for progression-free survival in the univariate analysis according to the type of immune checkpoint inhibitors

A

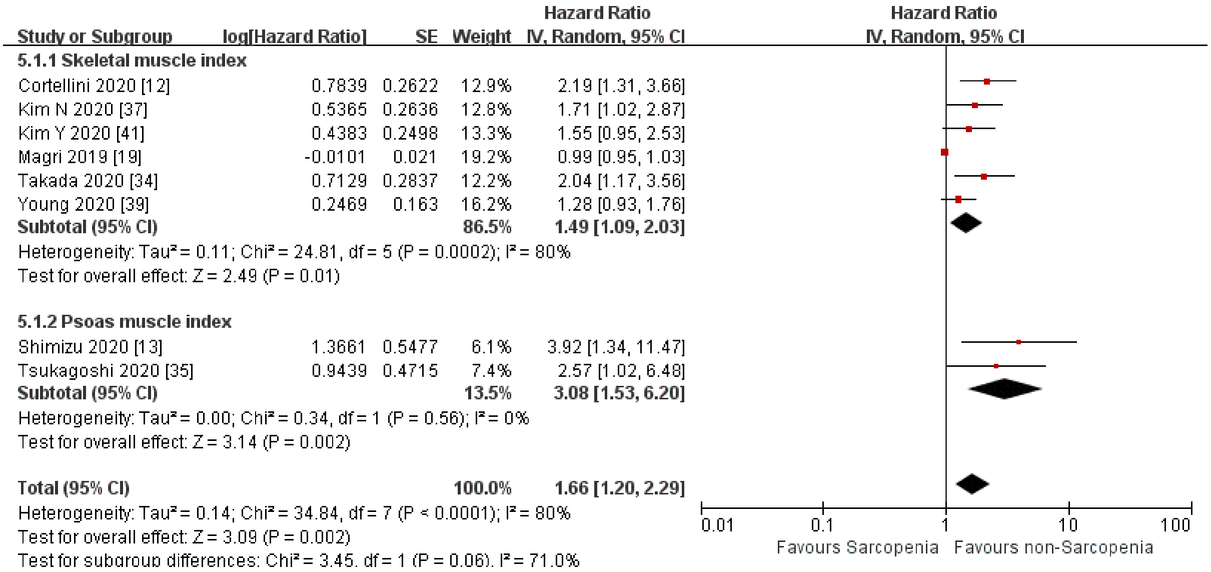

B

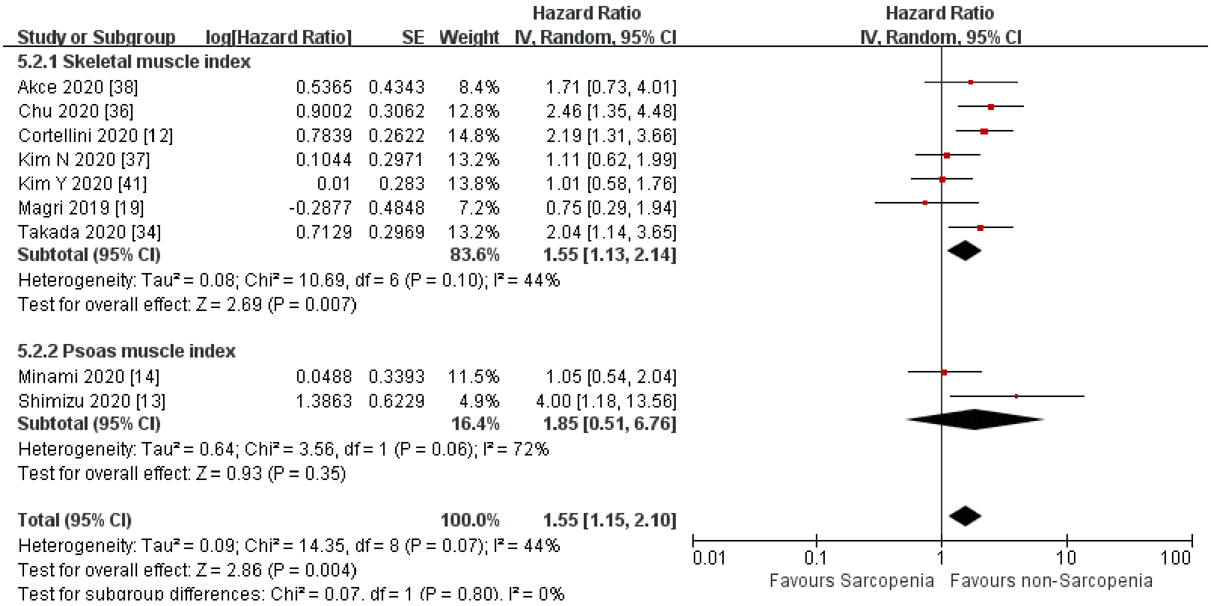

Supplementary Figure S5. Forest plots of hazard ratios for overall survival by skeletal muscle index and psoas muscle index—(**A**) overall survival in the univariate analysis, (**B**) overall survival with adjusted HRs.

A

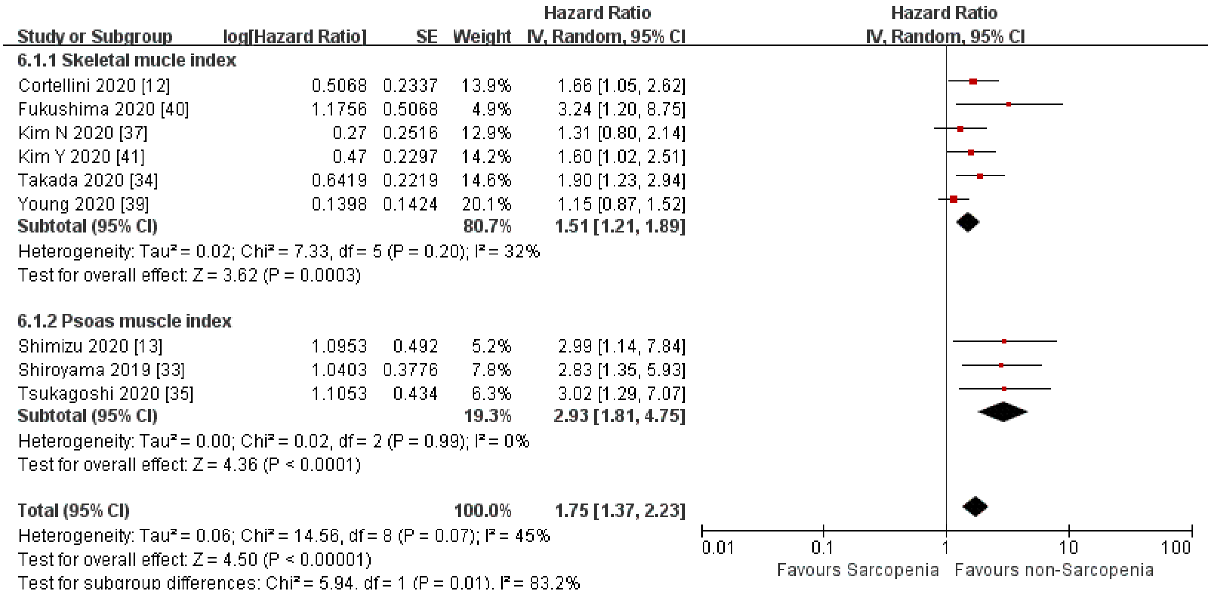

B

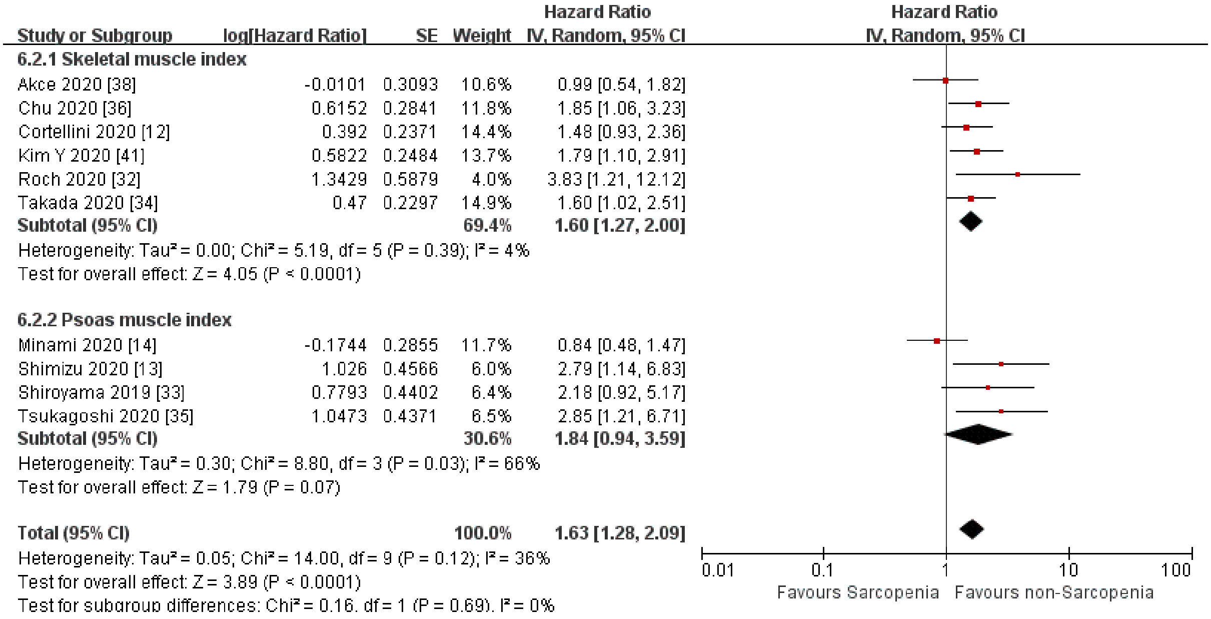

Supplementary Figure S6. Forest plots of hazard ratios for progression-free survival by skeletal muscle index and psoas muscle index—(**A**) progression-free survival in the univariate analysis, (**B**) progression-free survival with adjusted HRs.

Supplementary Table S1. Search strategy.

Categoy 1	Category 2	Category 3	Category 4	Category 5
muscle	immunotherap*	cancer	human	computed tomography
myosteatosis	PD1	tumor (tumo?r)	survival	CT
radiodensity	PD-1	malignancy (malignan*)	prognosis (prognos*)	MRI
skeletal muscle radiation attenuation	PD-L1	neoplasm (neoplas*)	complications (complicat*)	dual-energy X-ray absorption
cachexia (cache*)	PDL1	carcinoma (carci*)	cox	dual-energy X-ray absorptiometry
sarcopenia (sarcopen*)	PDCD1	malignant neoplasm (Emtree)	hazard ratio	DEXA
muscle depletion (muscle*)	CD274	neoplasm (Emtree)	prospective	DXA
muscle loss (muscle*)	programmed cell death	neoplasms (MeSH)	clinical trial	bioelectrical impedance analysis
muscle wasting (muscle*)	programmed death ligand		observational	BIA
muscle reduction (muscle*)	nivolumab		retrospective	computed tomography scanner (Emtree)
muscle strength (muscle*)	pembrolizumab		survival (Emtree)	computer assisted tomography (Emtree)
muscle mass (muscle*)	atezolizumab		survival rate (Emtree)	dual energy X ray absorptiometry (Emtree)
reduced muscle (muscle*)	avelumab		survival time (Emtree)	nuclear magnetic resonance imaging (Emtree)
depleted muscle (muscle*)	durvalumab		event free survival (Emtree)	spiral computed tomography (MeSH)
skeletal muscle (muscle*)	cemiplimab		long term survival (Emtree)	magnetic resonance imaging (MeSH)
muscle attenuation (muscle*)	immune checkpoint*		overall survival (Emtree)	
loss of muscle (muscle*)	checkpoint blockade*		disease free survival (Emtree)	
loss of skeletal muscle (muscle*)	"Immunotherapy"[Mesh]		cancer survival (Emtree)	
cachexia (Emtree)	"Nivolumab"[Mesh]		median survival time (Emtree)	
malnutrition (Emtree)	"pembrolizumab" [Supplementary Concept]		survival prediction (Emtree)	
skeletal muscle (Emtree)	"atezolizumab" [Supplementary Concept]		recurrence free survival (Emtree)	
muscle atrophy (Emtree)	"avelumab" [Supplementary Concept]		progression free survival (Emtree)	
muscle strength (Emtree)	"durvalumab" [Supplementary Concept]		prognosis (Emtree)	
sarcopenia (Emtree)	cemiplimab [Supplementary Concept]		cancer prognosis (Emtree)	
muscle mass (Emtree)	"Ipilimumab"[Mesh]		prospective study (Emtree)	
body composition (Emtree)			retrospective study (Emtree)	
muscle (Emtree)			clinical trial (Emtree)	
muscle function (Emtree)			observational method (Emtree)	
skeletal muscle (MeSH)			observational study (Emtree)	
sarcopenia (MeSH)			clinical outcome (Emtree)	
muscle strength (MeSH)			clinical outcome (Emtree)	
cachexia (MeSH)			survival rate (MeSH)	
muscles (MeSH)			survival (MeSH)	
			progression-free survival (MeSH)	
			survival analysis (MeSH)	
			prognosis (MeSH)	
			prospective studies (MeSH)	
			clinical trial (MeSH)	

	observational study (MeSH)<br>retrospective studies (MeSH)<br>humans (MeSH)

Supplementary Table S2. Search query and number of items found.

Search	Query	Items Found
	1. PubMed	
#16	(((((muscle* OR myosteatosis OR radiodensity OR skeletal muscle* radiation attenuation OR cachexia OR sarcopenia OR muscle* depletion OR muscle* loss OR muscle* wasting OR muscle* reduction OR muscle* strength OR muscle* mass OR reduced muscle* OR depleted muscle* OR skeletal muscle* OR muscle* attenuation OR loss of muscle* OR loss of skeletal muscle*) OR (skeletal muscle [MeSH] OR sarcopenia [MeSH] OR muscle strength [MeSH] OR cachexia [MeSH] OR muscles [MeSH])) AND ((immunotherap* OR PD1 OR PD-1 OR PD-L1 OR PDL1 OR PDCD1 OR CD274 OR programmed cell death OR programmed death ligand OR nivolumab OR pembrolizumab OR atezolizumab OR avelumab OR durvalumab OR cemiplimab OR immune checkpoint* OR checkpoint blockade*) OR ("Immunotherapy"[Mesh] OR "Nivolumab"[Mesh] OR "pembrolizumab" [Supplementary Concept] OR "atezolizumab" [Supplementary Concept] OR "avelumab" [Supplementary Concept] OR "durvalumab" [Supplementary Concept] OR cemiplimab [Supplementary Concept] OR "Ipilimumab"[Mesh]))) AND ((Cancer* OR tumor* OR malign* OR neoplasm* OR carcinoma) OR (neoplasms [MeSH]))) AND ((human OR survival OR prognos* OR complicat* OR cox OR hazard ratio OR prospective OR clinical trial OR observational OR retrospective) OR (survival rate [MeSH] OR survival [MeSH] OR progression-free survival [MeSH] OR survival analysis [MeSH] OR prognosis [MeSH] OR prospective studies [MeSH] OR clinical trial [MeSH] OR "Observational Study" [Publication Type] OR retrospective studies [MeSH] OR humans [MeSH]))) AND ((computed tomography OR CT OR MRI OR dual-energy X-ray absorption OR dual-energy X-ray absorptiometry OR DEXA OR DXA OR bioelectrical impedance analysis OR BIA) OR (spiral computed tomography [MeSH] OR magnetic resonance imaging [MeSH]))	279
#15	(computed tomography OR CT OR MRI OR dual-energy X-ray absorption OR dual-energy X-ray absorptiometry OR DEXA OR DXA OR bioelectrical impedance analysis OR BIA) OR (spiral computed tomography [MeSH] OR magnetic resonance imaging [MeSH])	1,381,178
#14	spiral computed tomography [MeSH] OR magnetic resonance imaging [MeSH]	483,677
#13	computed tomography OR CT OR MRI OR dual-energy X-ray absorption OR dual-energy X-ray absorptiometry OR DEXA OR DXA OR bioelectrical impedance analysis OR BIA	1,381,178
#12	(human OR survival OR prognos* OR complicat* OR cox OR hazard ratio OR prospective OR clinical trial OR observational OR retrospective) OR (survival rate [MeSH] OR survival [MeSH] OR progression-free survival [MeSH] OR survival analysis [MeSH] OR prognosis [MeSH] OR prospective studies [MeSH] OR clinical trial [MeSH] OR"Observational Study" [Publication Type] OR retrospective studies [MeSH] OR humans [MeSH])	21,100,508
#11	survival rate [MeSH] OR survival [MeSH] OR progression-free survival [MeSH] OR survival analysis [MeSH] OR prognosis [MeSH] OR prospective studies [MeSH] OR clinical trial [MeSH] OR "Observational Study" [Publication Type] OR retrospective studies [MeSH] OR humans [MeSH]	19,214,642
#10	human OR survival OR prognos* OR complicat* OR cox OR hazard ratio OR prospective OR clinical trial OR observational OR retrospective	21,083,571
#9	(Cancer* OR tumor* OR malign* OR neoplasm* OR carcinoma) OR (neoplasms [MeSH])	4,904,022
#8	neoplasms [MeSH]	3,436,486
#7	Cancer* OR tumor* OR malign* OR neoplasm* OR carcinoma	4,562,599
#6	(immunotherap* OR PD1 OR PD-1 OR PD-L1 OR PDL1 OR PDCD1 OR CD274 OR programmed cell death OR programmed death ligand OR nivolumab OR pembrolizumab OR atezolizumab OR avelumab OR durvalumab OR cemiplimab OR immune checkpoint* OR checkpoint blockade*) OR ("Immunotherapy"[Mesh] OR "Nivolumab"[Mesh] OR "pembrolizumab" [Supplementary Concept] OR "atezolizumab" [Supplementary Concept] OR "avelumab" [Supplementary Concept] OR "durvalumab" [Supplementary Concept] OR cemiplimab [Supplementary Concept] OR "Ipilimumab"[Mesh])	837,441
#5	"Immunotherapy"[Mesh] OR "Nivolumab"[Mesh] OR "pembrolizumab" [Supplementary Concept] OR "atezolizumab" [Supplementary Concept] OR "avelumab" [Supplementary Concept] OR "durvalumab" [Supplementary Concept] OR cemiplimab [Supplementary Concept] OR "Ipilimumab"[Mesh]	290,353
#4	immunotherap* OR PD1 OR PD-1 OR PD-L1 OR PDL1 OR PDCD1 OR CD274 OR programmed cell death OR programmed death ligand OR nivolumab OR pembrolizumab OR atezolizumab OR avelumab OR durvalumab OR cemiplimab OR immune checkpoint* OR checkpoint blockade*	623,529
#3	(muscle* OR myosteatosis OR radiodensity OR skeletal muscle* radiation attenuation OR cachexia OR sarcopenia OR muscle* depletion OR muscle* loss OR muscle* wasting OR muscle* reduction OR muscle* strength OR muscle* mass OR reduced muscle* OR depleted muscle* OR skeletal muscle* OR muscle* attenuation OR loss of muscle* OR loss of skeletal muscle*) OR (skeletal muscle [MeSH] OR sarcopenia [MeSH] OR muscle strength [MeSH] OR cachexia [MeSH] OR muscles [MeSH])	1,196,987
#2	skeletal muscle [MeSH] OR sarcopenia [MeSH] OR muscle strength [MeSH] OR cachexia [MeSH] OR muscles [MeSH]	722,190

#1	muscle* OR myosteatosis OR radiodensity OR skeletal muscle* radiation attenuation OR cachexia OR sarcopenia OR muscle* depletion OR muscle* loss OR muscle* wasting OR muscle* reduction OR muscle* strength OR muscle* mass OR reduced muscle* OR depleted muscle* OR skeletal muscle* OR muscle* attenuation OR loss of muscle* OR loss of skeletal muscle*	1,006,778
	2. Embase	
#16	#3 AND #6 AND #9 AND #12 AND #15	1,424
#15	#13 OR #14	2,443,915
#14	computed tomography scanner'/exp OR 'computer assisted tomography'/exp OR 'dual energy x ray absorptiometry'/exp OR 'nuclear magnetic resonance imaging'/exp	1,939,830
#13	computed tomography' OR 'ct' OR 'mri' OR 'dual-energy x-ray absorption' OR 'dual-energy x-ray absorptiometry' OR 'dexa' OR 'dxa' OR 'bioelectrical impedance analysis' OR 'bia'	1,590,600
#12	#10 OR #11	25,228,057
#11	survival'/exp OR 'survival rate'/exp OR 'survival time'/exp OR 'event free survival'/exp OR 'overall survival'/exp OR 'disease free survival'/exp OR 'cancer survival'/exp OR 'median survival time'/exp OR 'survival prediction'/exp OR 'recurrence free survival'/exp OR 'progression free survival'/exp OR 'prognosis'/exp OR 'cancer prognosis'/exp OR 'prospective study'/exp OR 'retrospective study'/exp OR 'clinical trial'/exp OR 'observational method'/exp OR 'observational study'/exp OR 'clinical outcome'/exp	4,518,116
#10	human' OR 'survival' OR 'prognos*' OR 'complicat*' OR 'cox' OR 'hazard ratio' OR 'prospective' OR 'clinical trial' OR 'observational' OR 'retrospective'	25,213,281
#9	#7 OR #8	6,885,293
#8	neoplasm'/exp	5,197,056
#7	cancer*' OR 'tumor*' OR 'malign*' OR 'neoplasm*' OR 'carcinoma'	6,356,934
#6	#4 OR #5	350985
#5	immunotherapy'/exp OR 'nivolumab'/exp OR 'pembrolizumab'/exp OR 'atezolizumab'/exp OR 'avelumab'/exp OR 'durvalumab'/exp OR 'cemiplimab'/exp OR 'immune checkpoint'/exp	257,909
#4	immunotherap*' OR 'pd1' OR 'pd-1' OR 'pd-l1' OR 'pdl1' OR 'pdcd1' OR 'cd274' OR 'programmed cell death' OR 'programmed death ligand' OR 'nivolumab' OR 'pembrolizumab' OR 'atezolizumab' OR 'avelumab' OR 'durvalumab' OR 'cemiplimab' OR 'immune checkpoint*' OR 'checkpoint blockade*'	321,812
#3	#1 OR #2	2,090,357
#2	cachexia'/exp OR 'malnutrition'/exp OR 'skeletal muscle'/exp OR 'muscle atrophy'/exp OR 'muscle strength'/exp OR 'sarcopenia'/exp OR 'muscle mass'/exp OR 'body composition'/exp OR 'muscle'/exp OR 'muscle function'/exp	1,287,127
#1	muscle*' OR 'myosteatosis' OR 'radiodensity' OR 'skeletal muscle* radiation attenuation' OR 'cachexia' OR 'sarcopenia' OR 'muscle* depletion' OR 'muscle* loss' OR 'muscle* wasting' OR 'muscle* reduction' OR 'muscle* strength' OR 'muscle* mass' OR 'reduced muscle*' OR 'depleted muscle*' OR 'skeletal muscle*' OR 'muscle* attenuation' OR 'loss of muscle*' OR 'loss of skeletal muscle*'	1,737,801
	3. Cochrane Central	
#1	muscle* OR myosteatosis OR radiodensity OR skeletal muscle* radiation attenuation OR cachexia OR sarcopenia OR muscle* depletion OR muscle* loss OR muscle* wasting OR muscle* reduction OR muscle* strength OR muscle* mass OR reduced muscle* OR depleted muscle* OR skeletal muscle* OR muscle* attenuation OR loss of muscle* OR loss of skeletal muscle*	
#2	MeSH descriptor: [Muscle, Skeletal] explode all trees	
#3	MeSH descriptor: [Sarcopenia] explode all trees	
#4	MeSH descriptor: [Muscle Strength] explode all trees	
#5	MeSH descriptor: [Cachexia] explode all trees	
#6	MeSH descriptor: [Muscles] explode all trees	
#7	#1 OR #2 OR #3 OR #4 OR $5 OR #6	
#8	immunotherap* OR PD1 OR PD-1 OR PD-L1 OR PDL1 OR PDCD1 OR CD274 OR programmed cell death OR programmed death ligand OR nivolumab OR pembrolizumab OR atezolizumab OR avelumab OR durvalumab OR cemiplimab OR immune checkpoint* OR checkpoint blockade*	
#9	MeSH descriptor: [Immunotherapy] explode all trees	
#10	MeSH descriptor: [Nivolumab] explode all trees	
#11	MeSH descriptor: [Ipilimumab] explode all trees	
#12	#8 OR #9 OR #10 OR #11	
#13	Cancer* OR tumor* OR malign* OR neoplasm* OR carcinoma	
#14	MeSH descriptor: [Neoplasms] explode all trees	
#15	#13 OR #14	
#16	human OR survival OR prognos* OR complicat* OR cox OR hazard ratio OR prospective OR clinical trial OR observational OR retrospective	
#17	MeSH descriptor: [Survival Rate] explode all trees	
#18	MeSH descriptor: [Survival] explode all trees	
#19	MeSH descriptor: [Progression-Free Survival] explode all trees	
#20	MeSH descriptor: [Survival Analysis] explode all trees	
#21	MeSH descriptor: [Prognosis] explode all trees	

#22	MeSH descriptor: [Prospective Studies] explode all trees
#23	MeSH descriptor: [Clinical Trial] explode all trees
#24	MeSH descriptor: [Observational Study] explode all trees
#25	MeSH descriptor: [Retrospective Studies] explode all trees
#26	MeSH descriptor: [Humanism] in all MeSH products
#27	#16 OR #17 OR #18 OR #19 OR $20 OR #21 OR #22 OR #23 OR #24 OR #25 OR #26
#28	computed tomography OR CT OR MRI OR dual-energy X-ray absorption OR dual-energy X-ray absorptiometry OR DEXA OR DXA OR bioelectrical impedance analysis OR BIA
#29	MeSH descriptor: [Tomography, Spiral Computed] explode all trees
#30	MeSH descriptor: [Magnetic Resonance Imaging] explode all trees
#31	#29 OR #30
#32	#7 AND #12 AND #15 AND #27 AND #31

Supplementary Table S3. Result types of the studies included in the meta-analysis.

Author / Year	OS		PFS	
	**Univariate Analysis**	**Adjusted HRs**	**Univariate Analysis**	**Adjusted HRs**
Cortellini 2020 [12]	V	V	V	V
Shimizu 2020 [13]	V	V	V	V
Minami 2020 [14]		V		V
Magri 2019 [19]	V	V		
Roch 2020 [32]				V
Shiroyama 2019 [33]			V	V
Takada 2020 [34]	V	V	V	V
Tsukagoshi 2020 [35]	V		V	V
Chu 2020 [36]		V		V
Kim N 2020 [37]	V	V	V	
Akce 2020 [38]		V		V
Young 2020 [39]	V		V	
Fukushima 2020 [40]			V	
Kim Y 2020 [41]	V	V	V	V
Total number of included studies	8	9	9	10

Supplementary Table S4. Covariates of the studies using adjusted HRs.

Author, year	Country	Cancer	Covariates
Cortellini 2020 [12]	Italy	NSCLC, Melanoma, RCC, others	Primary tumor, Number of metastatic sites, treatment line
Shimizu 2020 [13]	Japan	Urothelial carcinoma	neutrophil–lymphocyte ratio prognostic nutritional index ECOG, Liver metastasis
Minami 2020 [14]	Japan	NSCLC	serum albumin, neutrophil-to-lymphocyte ratio, performance status and driver mutations
Magri 2019 [19]	Italy	NSCLC	Sex, age, PS, Histology, smoking history, previous chemotherapy regimens, albumin, BMI, SMI, FFMI, FMI, weight change
Roch 2020 [32]	France	NSCLC	body mass index (BMI), the PD-L1 IHC score, the serum albumin level and the somatic genotype
Shiroyama 2019 [33]	Japan	NSCLC	Sex, ECOG PS
Takada 2020 [34]	Japan	NSCLC	Age, ECOG, Smoking history, Mutation status
Tsukagoshi 2020 [35]	Japan	NSCLC	Smoking history
Chu 2020 [36]	Canada	Melanoma	Age, male sex, LDH, positive BRAF status, ICI dosage
Kim N 2020 [37]	Korea	HCC	ECOG PS, ALBI group, AFP, Intrahepatic tumor burden, Surgery, RT, ALC & NLR risk group
Akce 2020 [38]	USA	HCC	Baseline CPS, Inflammation biomarkers: NLR for OS and PLR for PFS
Young 2020 [39]	USA	Melanoma	NA
Fukushima 2020 [40]	Japan	Urothelial carcinoma	NA
Kim Y 2020 [41]	Korea	Gastric cancer	previous chemotherapy regimens, Number of metastatic sites, ECOG PS, albumin, PD/SRC type [WD/MD], EBV negativity, HER2 negativity

NA: Not available
